# Early origins of mental disorder - risk factors in the perinatal and infant period

**DOI:** 10.1186/s12888-016-0982-7

**Published:** 2016-07-29

**Authors:** Louise Newman, Fiona Judd, Craig A. Olsson, David Castle, Chad Bousman, Penelope Sheehan, Christos Pantelis, Jeffrey M. Craig, Angela Komiti, Ian Everall

**Affiliations:** 1 The Centre For Women’s Mental Health, Royal Women’s Hospital, Parkville, Victoria 3015 Australia; 2 Department of Psychiatry, The University of Melbourne, Parkville, Victoria Australia; 3 Murdoch Children’s Research Institute, The Royal Children’s Hospital Melbourne, Parkville, Victoria Australia; 4 Department of Paediatrics, The University of Melbourne, Parkville, Victoria Australia; 5 Centre for Social & Early Emotional Development, School of Psychology, Deakin University, Geelong, Victoria Australia; 6 Psychiatry, St Vincent’s Hospital Melbourne, Fitzroy, Victoria Australia; 7 Faculty of Health Sciences, Australian Catholic University, Melbourne, Victoria Australia; 8 Department of General Practice, The University of Melbourne, Parkville, Victoria Australia; 9 Florey Institute of Neuroscience and Mental Health, The University of Melbourne, Parkville, Victoria Australia; 10 Centre for Human Psychopharmacology, Swinburne University of Technology, Hawthorn, Victoria Australia; 11 Department of Obstetrics and Gynaecology, Royal Women’s Hospital, Parkville, Victoria Australia; 12 Pregnancy Research Centre, The Royal Women’s Hospital, Parkville, Victoria Australia; 13 Department of Psychiatry, Melbourne Neuropsychiatry Centre, The University of Melbourne & Melbourne Health, Parkville, Victoria Australia; 14 NorthWest Mental Health, Melbourne Health, Parkville, Victoria Australia; 15 Department of Electrical and Electronic Engineering, Centre for Neural Engineering, The University of Melbourne, Parkville, Victoria Australia; 16 Department of Psychiatry, The Royal Melbourne Hospital, L1 North, Main Block, Parkville, VIC 3050 Australia

**Keywords:** Pregnancy, Babyhood, Attachment, Social and emotional development, Neurobiology

## Abstract

**Background:**

There is increasing understanding of the significance of early neurodevelopment in establishing risk for the range of mental disorders. Models of the early aetiology of mental disorders are complex with a range of potential factors from genetic and epigenetic to environmental influencing neurological and psychological development. Whilst the mechanisms are not fully understood, this paper provides an overview of potential biological and neurobiological factors that might be involved.

**Method:**

An aetiological model is presented and discussed. The discussion includes a range of risk factors for mental disorder. Maternal anxiety disorder is presented and reviewed as an example of the interaction of placental, epigenetic and early parenting factors elevating risk of poor neonatal outcome.

**Results:**

Available evidence points to the importance of in-utero influences as well as the role of early attachment and emotional care. Transgenerational mechanisms such as the impact of maternal mental disorder on foetal development are important models for examination of early risk. Maternal anxiety, as an example, is a significant risk factor for compromised mental health.

**Conclusions:**

Development of models for understanding the early origins of mental disorder is an important step in elaborating risk reduction strategies. Comprehensive early identification of risk raises the possibility of preventive interventions.

## Background

Early neurological development is central to current models of mental health and disorder. Understanding both intrinsic and environmental risk factors for mental disorder allows for the development of models of early intervention and prevention and more complex models of the emergence of disorder. There are currently several approaches to developing modelling such as the Fetal Programming Hypothesis [1, 2]. Developmental stage specific models of early development and risk have been proposed to assist understanding of the complex interplay between these factors and have more recently looked at integrative approaches [3]. The purpose of this paper is to outline an intergrative approach including (1) a model of early neurodevelopment and developmental risk and summarises evidence relating to the significance of early environment and experience on developmental pathways, and (2) uses maternal mental illness as an example to illustrate some of the complex mechanisms to be considered when modelling early developmental risk, including in utero and environmental factors and their interplay. Current understanding in the literature points to the significance of early development in emerging capacity for adaption and response to stress and the development of resilience on multiple levels. Current models of the concept of resilience focus not only on stress regulation mechanisms but also on the processes of flexible change in brain organisation and function and the implications of this for ongoing development. Mental health problems in the broadest sense may be seen as a variable negative outcome when there are difficulties in repair and homeostasis (for review see Russo et al [4]).

**Fig. 1:**
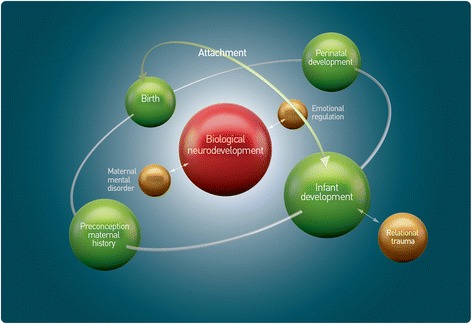
Interaction of early developmental domains

The perinatal period (in utero to twelve months) and infant (twelve months to three years) developmental periods are focussed on as the establishment of neurological and psychological capacities and functioning, which shapes developmental pathways and later mental health. There is increasing recognition that early development is shaped by the interaction of neurodevelopmental, biological and psychosocial factors and that developmental risk emerges on all or any of these levels. Figure 1 illustrates the interaction of factors involved in early development.

One integrated approach model of risk is the developmental origins of health and disease approach (DOHaD) [5], which suggests that adaptation by the developing foetus to a detrimental in-utero environment leads to changes in early brain structure and function. A key assumption is that biological systems undergo rapid developmental changes and are therefore especially vulnerable to both organising and disorganising influences. The brain’s extended pre- and post-developmental trajectory means that early life experiences have the potential to sculpt brain morphology. The sculpting of the immature brain is an interactive process between genetic programming, cell function and the environment. Proper timing and guidance of neurogenesis, neuronal differentiation, migration, apoptosis, synaptogenesis, myelination and synaptic pruning are critical to the emerging organisation and functioning of the brain [6]. The quality of early emotional interaction and the context of attachment relationships shape early development. Specifically the quality of the caregiving relationship shapes both neurological and psycho-social development [6–8]. The infant developmental period is one of rapid neurological organisation and is the period when pathways underlying core psychological functions are laid down [9], Adversity and trauma in infancy including maltreatment and disruption of attachment relationships are seen as significant developmental risk factors and increase vulnerability to a range of later onset mental disorders [10]. The concept of “neuro-vulnerability” to mental disorder focuses on the interaction of risk at several levels (genetic, epigenetic, in utero, perinatal and infant experience) and is important in thinking about the early origins of mental disorder regardless of age of presentation or emergence of symptoms.

Infant brain development is fundamentally shaped by the quality of interaction with the primary carer [11]. The development of neural pathways, synapses and emergent neurological functioning develops in respect to direct input, a process which has been described as being ‘experience-dependent’ [8]. Parenting and attachment relationships are the social context of early brain development and essential processes such as stress regulation and HPA axis functioning, which mediates on- going risk and vulnerability [12].

Current theoretical understanding focuses on the integration of developmental domains, including neurological and psychosocial models. Human development is increasingly understood as being shaped by social experience regardless of the examined domains, moving away from previous models which focussed on developmental stages [13, 14]. Current developmental approaches also focus on the direct contribution of the infant to relational functioning and their capacity to influence environmental responses. This is reflected in the work of Sameroff [15, 16] who describes the developmental transaction between the child and social experiences. This type of modelling has influenced much of the current infant developmental research, which is attempting to describe the evolution of adaptive and maladaptive developmental trajectories and the factors in both parent and child which contribute to developmental outcome [17].

Attachment Theory in more recent iterations focuses on disturbances of social interaction and the implication of this for both neurological and psychological development. Specifically, there is an increasing focus on the potential impact of relational stress and trauma on neurological development [18, 19]. There is also increasing understanding of the interaction between intrinsic risk factors and the quality of the social environment and specifically the role of relational ‘trauma’ in the infant period [20].

The field of developmental psychopathology focuses on both the understanding of the impact of disorder on development as well as the way in which disorders manifest in different developmental stages. This has allowed for more complex developmental understanding of specific disorders including psychotic conditions and personality disorders [21]. A more complex model of developmental risk has emerged which allows closer examination of distorted interactions and traumatic experiences in infancy and the way in which relationship patterns may be transmitted across generations [22–24].

## A high risk example - children of women with maternal anxiety

The impact of maternal mental illness on infant development and developmental risk has been studied from both neurodevelopmental and in utero factors and psychosocial impacts on parenting and infant experience, with ongoing discussion about the role of both levels and their interaction.

Amongst mothers with mental illness, most work has focused on maternal anxiety (variously measured by self-report instruments) during pregnancy. However, the mechanisms underlying the association between maternal anxiety and infant developmental outcomes remain obscure. Focus on maternal anxiety allows for a better understanding of the multiple effects on development including the neurodevelopmental impact of stress related hormones as well as the psychosocial impact on parenting behaviour. This focus is central to the development of intergrative models of development of psychopathology.

Maternal anxiety during pregnancy has been linked to problems of infant temperament, behaviour, and cognitive development; emotional and behavioural problems in children and adolescents; and structural brain changes (see Table 1 for examples of key studies). However, it is evident that such problems cannot be attributed solely to the in utero influence of maternal mood but must also be influenced, by a range of factors from birth onwards. Environment and contextual factors such as exposure to conflict and interpersonal violence, social deprivation and dislocation such as experienced by asylum seekers, clearly are important in understanding the impact on maternal mental health and the neurodevelopment of the child. It should be noted however many studies report associations rather than causal connections between maternal anxiety and adverse outcomes and thus further work is required to elucidate causal pathways.

**Table 1:** Effects of maternal antenatal stress and anxiety on outcomes for infants and children

Infant and child outcomes	
Infant temperament	
• Reduced attention regulation at 3 and 8 months [1] • Greater child fearfulness scores 14–19 months [2]	
Development and cognitive functioning	
• Lower Bayley Scales Mental Development Index (MDI) and Psychosocial Development Index (PDI) scores at 8 months [3] • Lower Bayley Scales MDI scores at 1–2 years [4] • Lower productive language scores at 2 years [5] • Poorer verbal intelligence and language skills at 5 ½ years [6] • Lower inhibitory control at 6–9 years [7] • Impulsive response pattern on cognitive tasks at 14/15 years [8]	
Emotional and behavioural problems	
• Greater total problem behaviour at 27 months, 4 years and 81 months [9–11] • Greater hyperactivity/inattention in boys at 8–9 years [12] • Greater externalising problems 8–9 years [13] • Greater anxiety aged 6–9 years [14] • Greater internalising difficulties in 7–8 years [15] • Greater depressive symptoms in adolescence [16]	
Structural brain changes	
• Reduced head circumference corrected for birth weight [17, 18] • Reduced grey matter in prefrontal cortex, medial and lateral temporal lobe, postcentral gyrus and cerebellum on MRI scan at 6–9 years [19]	

## Mechanisms of neurovulnerability

### Developmental origins

The mechanisms underlying the association between maternal anxiety and developmental outcomes are not yet fully understood. Several mechanisms may contribute to these in utero effects including the influence of maternal stress-related hormones on foetal development, epigenetic changes and direct impact on foetal brain structures and stress regulation mechanism. These vulnerabilities are then amplified or contained in the context of infant experience and quality of care, particularly the capacity of the parent to support infant regulation of anxiety and emotional states.

### Placental mechanisms

Maternal stress hormones, particularly cortisol, may have effects on a vital organ of foetal development, the placenta. The placenta plays an important role in protecting the foetus from the mother’s cortisol through the expression of the inactivating enzyme, 11beta hydroxysteroid dehydrogenase type 2 (11β-HSD2); hence, any changes in enzyme expression in this pathway may leave the foetus vulnerable to the mother’s circulating hormones. One recent study has shown an association between maternal trait and state anxiety and reduced placental 11β-HSD2 expression, suggesting a possible mechanism for excessive foetal cortisol exposure in women with maternal anxiety [25]. This is consistent with a further study showing increased placental 11β-HSD2 methylation following exposure to maternal anxiety [26]. However, increased 11β-HSD2 has also been associated with maternal depression and anxiety as well as increases in the placental human serotonin transporter [27]. These opposing findings may represent methodological differences in patient selection and the diagnosis of anxiety in the small amount of preliminary data available. For example, in the first expression study [25] patients were recruited prior to elective Caesarean Section without any regard for a previous mental health diagnosis and the results were analysed on the basis of questionnaires administered at the time of recruitment. The latter expression study [27] recruited patients on the basis of a clinical diagnosis of anxiety but without any degree of standardisation or quantification by validated questionnaire. Such methodological discrepancies highlight the need for more detailed studies evaluating the effects of maternal anxiety on the complex cortisol pathway in the placenta.

### Neurodevelopmental mechanisms

Maternal anxiety during pregnancy may play a critical role in the development of foetal brain structures important to regulation of the infant stress response and social cognition and engagement. This is a new area of research, and one that is yielding some important insights into very early development. For example, juvenile and adult rats exposed to prenatal stress have been shown to have decreased numbers of glucocorticoid receptors in the hippocampus [28] and decreased spine density in the anterior cingulate gyrus and orbitofrontal cortex [29]. Newborns of human mothers who experienced high anxiety during their second trimester of pregnancy have greater right frontal EEG activation, a physiological profile that has been linked to depression in adults [30]. A structural MRI study of children aged 6–9 years has revealed important relationships between maternal gestational anxiety (second trimester) and volume reductions in multiple brain regions, including the prefrontal cortex, medial temporal lobe (including areas connected to the hippocampus), cerebellum and post-central gyrus [31] as well as impairments in executive function [32].

Foetal exposure to maternal stress hormones, in particular glucocorticoids transmitted across the placenta, may play an important role in foetal neurodevelopment. Foetal and maternal plasma concentrations of cortisol, testosterone, and to a lesser extent, corticotrophin-releasing hormone (CRH) are correlated [33–35], although levels are higher (~10-fold) in mothers. Foetal exposure to maternal cortisol and CRH has been associated with reduced physical and neuromuscular maturation in newborns [36]. Animal studies have shown that administration of glucocorticoids to pregnant rats significantly alters neuronal structure and synapse formation and inhibits neurogenesis in offspring [37]. Further, a study in humans found that maternal cortisol levels in early pregnancy were associated with larger amygdala (but not hippocampal) volumes and higher levels of affective symptoms in girls at 7 years of age [38]. Human foetal exposure to exogenous glucocorticoids (dexamethasone) has also been associated with increased internalising problems in childhood [39].

### Gene by environment mechanisms

Examinations of gene-environment mechanisms typically follow one of two dominant frameworks: (1) diathesis-stress framework [40] or (2) differential susceptibility framework [41]. The diathesis-stress framework has guided the majority of gene-environment studies to date and postulates an individual’s vulnerability is dependent on the biological context (e.g. genotype) in which adversity (e.g. prenatal maternal anxiety, childhood abuse) is encountered. However, this traditional approach has more recently been challenged by the differential susceptibility framework, which suggests an individual’s biological context moderates sensitivity to both negative and positive environmental influences and views traditionally labelled ‘vulnerable’ individuals as “plastic/malleable’ individuals.

The serotonin transporter (*SLC6A4*) gene polymorphism (HTTLPR) is arguably the most studied gene for its interactions with various adverse and supportive environments, albeit genes involved in HPA-axis function (e.g. *NR3C1, FKBP5*) are also widely studied. The landmark paper by Caspi et al. [42] reported that young adults with the HTTLPR short (*s*) allele were more likely to develop depression after exposure to severe childhood abuse compared to those with the long (*l)* allele and proposed that the *s* allele may be a moderator (effect modifier) of the relationship between stress and depression. Since the publication of the Caspi study, similar results have been demonstrated. In a large Dutch cohort (*n* = 1513) infants who carried the *s* allele were more negatively emotional when mothers reported anxiety during pregnancy compared to *l/l* genotype carriers [43]. Whereas, Eley et al. [44] showed the *s/s* genotype conferred more susceptibility to depression among female adolescents in high stress environments but *s/s* genotype carriers in low-stress environments were less likely to be depressed, suggesting the s allele confer sensitivity rather than vulnerability to environmental factors. Nevertheless, the current evidence shows support for both gene-environment frameworks. Future integration of these frameworks with the likely underlying epigenetic mechanisms will expand our understanding of the interplay between our genes and our environments.

### Epigenetic mechanisms

Epigenetic modifications are another mechanism that may explain how an adverse intra-uterine environment can be associated with disease many years after exposure. ‘Epigenetic’ refers to mitotically-heritable alterations in gene function in the absence of changes in the DNA sequence. Examples of epigenetic alterations include DNA methylation, histone modifications and non-coding RNAs. It is likely that stress related hormones (cortisol) originating from the mother play a crucial role. These hormones can cross the placental barrier into the foetal blood and if present in abnormal amounts during specific sensitive developmental periods may disturb the programming of certain biological systems responsible for the regulation of foetal development and later behaviour. For example, animal and human studies have shown that DNA methylation at birth in the cortisol-binding glucocorticoid receptor (*NR3C1*) and the cortisol-inactivating enzyme 11beta hydroxysteroid dehydrogenase type 2 (*11β-HSD2*) - genes central to the stress response pathway - can be regulated by maternal anxiety and depression [26, 45–47]. Furthermore, methylation levels at these genes correlated with poor stress response [45] or neurodevelopment [26] in infants. There is also evidence that stress in early life has lasting effects on methylation at NR3C1 in animals [28, 48] and humans [49] and at least in animal models, such effects appear to be reversible by diet [50] or pharmaceutical [51] interventions.

Cortisol is essential for normal brain development, but exposure to excessive amounts has long-lasting effects on neuroendocrine functioning and on behaviour. Cortisol is known to have profound effects on the developing brain, it can modify cell proliferation and differentiation and synaptic development in various brain regions. Although brain change and adaptation are part of a lifelong process, the earliest phases of maturation during foetal development and early childhood are arguably the most dramatic and important in terms of laying the foundations for core structures and functions and developmental risk. The challenge of contemporary developmental epigenetics is to identify sensitive periods in early development with increasing precision. To do this new studies that assess the methylome at multiple time points from conception to early childhood are needed. Such studies will provide new insights into how methylome changes in response to specific features of the physical and social environment in early life. This will present new opportunities to develop neuroprotective interventions in infancy to reduce the burden of altered brain growth and poor functional and behavioural outcomes.

### Attachment and caregiving mechanisms

Parenting and the quality of care is increasingly recognised as shaping brain structure and function [52, 53]. Contemporary attachment theory has more recently shifted its’ focus to clinical populations. This focus on clinical populations and the way in which parental mental disorder contributes to disturbances in early interactions allows for further examination of early neurodevelopment and its’ possible relationship to later disorder [18, 19]. Early experiences of care and emotional interaction are increasingly understood as impacting directly on brain growth and organisation with recognition that early attachment relationships can set up both resilience and risk for mental disorder. Specifically, emotional and stress regulation occur in a dyadic context and infant neurological organisation is shaped by the experience with the caregiver [53].

The infant brain grows in direct response to patterns of stimulation and activation. The quality of early emotional care and emotional regulation in the context of caregiving relationships shapes brain organisation and the emergence of core neuropsychological capacities such as affect regulation, attention, stress regulation and interpersonal functioning and are essential for later psychological functioning. The question of the significance of early neurodevelopmental disruption is a crucial one in current understanding of mental disorder with most literature focused on the implications of early trauma in terms of neurovulnerability to the range of mental disorders.

The influence of poor care, child abuse and maltreatment suggests complex effects on brain development related to elevated levels of catecholamines, delays in myelination and abnormalities in neuronal pruning [54–56]. Less well documented are the impacts of specific maternal mental disorders on child neurodevelopment and the specific mechanisms involved. Postnatal depression remains the most studied maternal disorder and is associated with child depressive symptoms, cognitive impairment most severe in male infants, and anxious attachment [56–58]. Maternal anxiety will also impact on the development of parent’s self-concept and sense of efficacy both immediately after the birth, with the stress of becoming a new mother, as well as longer-term good parenting skills. Anxious parents are more likely to experience parenting related stress and to have difficulties in establishing secure attachment relationships with their infants. Maternal anxiety is associated with infant attachment insecurities (i.e., anxious, ambivalent and disorganized), stress and behavioural dysregulation, and longer-term vulnerability to emotional and mental disorder [59–61].

Trauma in infancy has been defined functionally as those experiences resulting in high levels of stress related hormones and ongoing experiences of unresolvable fear and anxiety, most commonly in the context of aberrant interactions of the primary carer. The carer may fail to respond to infant affective communication or respond in a confusing or distorted manner resulting in infant anxiety and if persistent, a state of disorganisation of attachment which is associated with cortisol dysregulation and negative impact on the development of self-regulation and interpersonal functioning.

Severe trauma in infancy such as maltreatment and abuse has emerged as a non-specific risk-factor for the range of mental disorders suggesting that it contributes to neurovulnerability that is likely moderated by genetic variation [4] and mediated by factors such as type and frequency of early adverse experiences and the developmental period [62]. Whilst early trauma increases the overall risk of mental disorder (dose effect), the relationships between early trauma and specific diagnoses are complex and influenced by severity of adversity as well as age of onset. Borderline personality disorder was found to be specifically associated with early onset of adversity and early adversity to be predictive of affective dysregulation, and later depression.

### Transgenerational mechanisms

Attachment theory has come to elaborate the way in which the parent’s own early history of attachment and attachment related trauma influences parenting capacity and understanding of infant social communication [63]. The infant’s experience of the parent develops in part as a result of the parent’s own history and early experiences. Assessment of the quality of parental socio-emotional interaction with the infant is based on the understanding of the adult’s own early experiences [64–66]. Further, parenting behaviour and core components such as the ability to respond in an appropriate way to an infant’s attempts at communication is influenced by brain pathways shaping affiliate behaviour and emotional processing [53].

## Implications for research and clinical practice

Research examining complex interactional developmental models is sparse. Longitudinal studies of risk factors commencing in utero and following psychosocial and environmental factors are needed to gain further understanding of the relative contribution of risk factors and their relationship to mental health outcomes. New studies are now following the offspring of established cohort studies, opening opportunities to investigate transgenerational effects.

Clinical interventions aimed at improving maternal mental health and functioning and infant outcome may be described as ‘neuroprotective’ and have the potential to reduce the risk of mental disorder. Infant developmental outcome is related to maternal quality of care and sensitivity and the capacity to focus on the infant’s affective communication. Current interventions broadly aim to improve maternal response to the infant’s emotional and social cues and to support the parents in their developing understanding of the infant’s psychological states and needs. Parents with mental health issues such as depression, anxiety and psychosis may have difficulty in reading and ‘interpreting’ the communication of the infant and may show patterns of mistimed and misattuned interactions, which increase infant stress and are associated with attachment disorganisation. Longitudinal studies of infants in this group have found increased in stress related symptoms in adolescent suggesting long term sequelae of early emotional trauma.

## Conclusions

Treatment of maternal anxiety disorder and stress related conditions during pregnancy are seen as central to protecting foetal development. Similarly, early intervention in disturbances of parent-infant emotional interaction and regulation and reduction of infant stress is a key component of intervention with potentially adverse developmental pathways. Early interaction approaches focus on improving the quality of parent-infant emotional regulation and building the parent’s capacity to read and respond to infant communication. Promotion of attachment organisation is seen as a core component of developmentally focussed preventive approaches such as the Circle of Security program [67] and the Mind the Baby approach [68]. Studies of infant stress and cortisol regulation in response to interactional interventions may be important in evaluating perinatal programs and elucidating the neurobiological underpinnings of psychological interventions.
